# Controlled Aggregation and Increased Stability of β-Glucuronidase by Cellulose Binding Domain Fusion

**DOI:** 10.1371/journal.pone.0170398

**Published:** 2017-01-18

**Authors:** Soo-Jin Yeom, Gui Hwan Han, Moonjung Kim, Kil Koang Kwon, Yaoyao Fu, Haseong Kim, Hyewon Lee, Dae-Hee Lee, Heungchae Jung, Seung-Goo Lee

**Affiliations:** 1 Synthetic Biology & Bioengineering Research Center, KRIBB, Yuseong-gu, Daejeon, Korea; 2 Department of Chemical Engineering and Applied Chemistry, Chungnam National University, Daejeon, Korea; 3 Biosystems & Bioengineering, University of Science & Technology, Yuseong-gu, Daejeon, Korea; Karl-Franzens-Universitat Graz, AUSTRIA

## Abstract

Cellulose-binding domains (CBDs) are protein domains with cellulose-binding activity, and some act as leaders in the localization of cellulosomal scaffoldin proteins to the hydrophobic surface of crystalline cellulose. In this study, we found that a CBD fusion enhanced and improved soluble β-glucuronidase (GusA) enzyme properties through the formation of an artificially oligomeric state. First, a soluble CBD fused to the C-terminus of GusA (GusA-CBD) was obtained and characterized. Interestingly, the soluble GusA-CBD showed maximum activity at higher temperatures (65°C) and more acidic pH values (pH 6.0) than free GusA did (60°C and pH 7.5). Moreover, the GusA-CBD enzyme showed higher thermal and pH stabilities than the free GusA enzyme did. Additionally, GusA-CBD showed higher enzymatic activity in the presence of methanol than free GusA did. Evaluation of the protease accessibility of both enzymes revealed that GusA-CBD retained 100% of its activity after 1 h incubation in 0.5 mg/ml protease K, while free GusA completely lost its activity. Simple fusion of CBD as a single domain may be useful for tunable enzyme states to improve enzyme stability in industrial applications.

## Introduction

Cellulose binding domains (CBDs), which are part of cellulose-degrading enzymes, are non-catalytic domains found mostly in cellulolytic microorganisms, typically as a domain on the scaffoldin protein of the multi-enzyme complex cellulosome [1]. CBDs have been reported to enhance enzyme activity by concentrating the enzyme on the substrate surface and/or disrupting non-covalent interactions between adjacent substrate molecules [2–4]. Previous studies by our group reported another role for family II CBDs from *Cellulomonas fimi* that induced the formation of active inclusion bodies (IBs) when fused with the target protein in the cytoplasm of *Escherichia coli* [5,6]. As a new matrix, CBD may be useful for enhancing enzyme properties and reducing the costs of enzyme catalysts.

β-Glucuronidase (GusA) is the most widely used reporter enzyme in studies using transgenic plants because it can easily be assayed using various commercially available chromogenic, colorimetric, and fluorometric substrates [7,8]. The *E*. *coli* GusA provides an excellent model system for studying directed evolution *in vitro* and determining structure–function relationships because of its efficiency, biosafety, scientific applications, cost-effectiveness, and commercial potential [9,10]. However, it shows a high proportion of false-positives because of endogenous GusA activity in the high-throughput screening processes. The solution for eliminating potential false-positives and increasing the accuracy of the screening system may be achieved by enhancing the stability of GusA [8].

Here, our main objective of this study is focused in the production of soluble GusA protein with improved stability using a CBD fusion. To characterize the physical properties of CBD from *C*. *fimi*, we attempted to obtain soluble GusA-CBD under low temperatures and low agitation for protein overexpression, and then successfully purified soluble CBD-fused enzymes. In this study, free GusA and GusA-CBD were cloned, expressed in soluble form, purified, characterized, and compared in terms of their biochemical properties, such as multimeric state, optimal pH and temperature, thermostability, pH stability, methanol tolerance, and protease K accessibility. The results revealed increased stability of GusA upon simple fusion of the CBD as a single domain. This is the first study to characterize the properties of family II CBDs *in vitro* after induction of a new fusion partner. Therefore, our results provide an alternative method for improving the catalytic performance of target enzymes such as GusA using CBD with desired catalytic performance.

## Materials and Methods

### Materials

*p*-Nitrophenyl β-d-glucuronide (*p*NPGA), and *p*-nitrophenol (pNP) were purchased from Sigma Chemicals (St. Louis, MO, USA).

### Microorganisms, plasmids, media, and culture conditions

*E*. *coli* MG1655, *E*. *coli* C2566 (New England Biolabs, UK), and the pET-21a (+) plasmid (Novagen, Darmstadt, Germany) were used as the source of genomic DNA for GusA, as host cells for protein expression, and as the expression vector, respectively. The *E*. *coli* C2566 harboring GusA or GusA-CBD were cultivated in 500 ml of Luria–Bertani (LB) medium in a 2,000-ml flask containing 50 μg of ampicillin/ml at 37°C with shaking at 250 rpm. When the optical density of the bacteria reached 0.6 at 600 nm, isopropyl-β-d-thiogalactopyranoside (IPTG) was added at a final concentration of 0.1 mM to induce expression of GusA or GusA-CBD enzymes, and the cultures were incubated with shaking at 150 rpm at 16°C for 16 h.

### Cloning of the genes encoding GusA, GusA-CBD and CBD-GusA

The *gusA* gene was amplified by PCR using genomic DNA isolated from *E*. *coli* MG1655 as a template. The CBD gene was obtained from the *C*. *fimi* KCTC9143 exo-1,4-β-D-glucanase gene. The sequences of the oligonucleotide primers used for cloning were designed using the published DNA sequence for GusA from *E*. *coli* MG1655. All primers were synthesized by Bioneer Co. (Daejeon, Korea). The forward and reverse primers for the *gusA* fragment were designed for introduction of the underlined *Nde*I and *Hind*III restriction sites, respectively. The DNA fragment for GusA-CBD and CBD-GusA were prepared using overlap PCR using forward and reverse primers, respectively and then they were prepared for introduction using the underlined *Nde*I and *Hind*III restriction sites, respectively. The amplified DNA fragments were extracted using a gel extraction kit (Promega, Madison, WI) and were cloned into the pGEM-T Easy vector (Promega). DNA sequencing was carried out with the service by Macrogen Co. (Seoul, Korea). The *Nde*I*-Hind*III fragment from the T-vector containing GusA-CBD and CBD-GusA were subcloned into the same site of the pET-21a (+) vector and transformed into the *E*. *coli* ER2566 strain.

### Purification of GusA, GusA-CBD and CBD-GusA

The recombinant cells were harvested and resuspended in buffer A (50 mM sodium monophosphate, 300 mM NaCl, 10 mM imidazole, and 0.1 mM phenylmethylsulfonyl fluoride (PMSF) as a protease inhibitor). The resuspended cells were disrupted by ultrasonication (Fisher Scientific, Pittsburgh, PA) on ice. The cell debris was removed by centrifugation at 15,000 × *g* for 20 min at 4°C and the supernatant was filtered through a 0.45 μm filter and applied to an IMAC chromatography column (Bio-Rad, Hercules, CA) equilibrated with buffer A. The bound protein was eluted with a linear gradient between 10 mM and 250 mM imidazole in buffer A. The active fraction was dialyzed in 50 mM HEPES buffer (pH 7.5) and the resulting solution was used as the purified enzyme. All purification steps using columns were carried out using a Profinia^™^ Affinity Chromatography Protein Purification System (Bio-Rad). The protein concentration was quantified by the method reported by Bradford[11]. The purified proteins were confirmed by SDS-PAGE gel analysis.

### Molecular mass determination

The molecular mass of GusA, GusA-CBD, and CBD-GusA were examined by SDS-PAGE under denaturing conditions, using a pre-stained ladder (Bio-Rad) as a reference proteins. All protein bands were stained with Coomassie blue for visualization. The molecular mass of the native enzymes were estimated using an HPLC system (Agilent 1260, Santa Clara, CA) equipped with a UV detector at 210 nm using a ZORBAX GF-250 (250 mm × 4.6 mm, Agilent). The column was initially eluted with 130 mM NaCl/20 mM Na_2_HPO_4_ (pH 7.0) as the mobile phase. The flow rate was 1.0 ml/min and the column temperature was 23°C. The column was calibrated with thyroglobulin (669 kDa), aldolase (158 kDa), albumin (67 kDa), ovalbumin (43 kDa) and chymotrypsinogen A (25 kDa), as reference proteins, and the molecular mass of the native enzyme was calculated by comparing with the migration length of reference proteins. The molecular mass of the aggregated native enzymes were estimated after 12 h using a Superose_12 10/300 column for gel filtration chromatography (Amersham Biosciences). The enzyme solution was applied to the column and eluted with 50 mM Tris-HCl (pH 7.5) buffer containing 150 mM NaCl at a flow rate of 1 ml/min.

### Enzymes assay

The catalytic activities of GusA, GusA-CBD, and CBD-GusA were determined based on the amount of *p*NP released from 0.5 mM *p*NPGA in 0.2 mL of HEPES buffer (pH 7.5) in a round-bottomed 2-ml tube, respectively. The GusA, GusA-CBD, and CBD-GusA enzyme reactions were conducted at 60°C, 65°C and 65°C, respectively, for 10 min and terminated by adding 0.1 M Na_2_CO_3_. After centrifugation for 15 min at 16,300 × *g* to clear the reaction solution, the absorbance change at 420 nm was measured using a Victor V Multilabel Plate Reader (PerkinElmer Life Sciences, Waltham, Massachusetts, USA). One unit of enzyme was defined as the activity required to produce 1 μmol of *p*NP as a product per min under the specified assay conditions.

### Effects of temperature, pH, methanol and protease K on the activity of GusA and GusA-CBD

To examine the effect of pH on the activities of GusA and GusA-CBD, the pH was varied between 4.5 and 8.5 using 50 mM sodium acetate buffer (pH 4.5–6.5), 50 mM potassium-phosphate buffer (pH 6.5–7.0), and 50 mM HEPES buffer (pH 7.0–8.5). To investigate the effect of temperature on the activities and stabilities of GusA and GusA-CBD, the temperature was varied from 45°C to 75°C. The experimental data for thermal deactivation of enzymes were fitted to a first-order curve, and the half-lives of the enzymes were calculated using the SigmaPlot 12.0 software (Systat Software, San Jose, CA). To examine the effect of methanol as an organic solvent on the activities of GusA and GusA-CBD, the methanol concentration was varied between 0% and 20%. The protease K accessibility was performed at different concentration of protease K for 1h, and then tested activity of GusA and GusA-CBD.

## Results

### Expression and purification

GusA-CBD and CBD-GusA were expressed in *E*. *coli* at a low temperature (16°C) after 0.1 mM IPTG induction and low speed of 150 rpm for the expression of soluble CBD protein. The crude extract obtained from harvested cells as a soluble protein was purified using a His-tag affinity chromatography column. Purified free GusA, GusA-CBD, and CBD-GusA showed specific activities of 102 ± 4.3, 19 ± 0.6, and 4.3 ± 0.1 units mg^–1^ (μmol mg^–1^ min^–1^), respectively. The molecular weights of purified free GusA, GusA-CBD, and CBD-GusA were determined using SDS-PAGE, showing molecular weights of approximately 68.5, 78, and 78 kDa, respectively (S1 Fig). These values were consistent with the calculated values determined using the Compute pI/Mw tool [12]. The expression level and specific activity of CBD-GusA decreased compared to those of GusA-CBD, suggesting that CBD may influence GusA expression and activity when fused at the N-terminus of the target protein. We therefore performed further characterization experiments of GusA-CBD and compared the results with the data of free GusA enzyme.

### Self-assembly of soluble GusA-CBD

**Fig 1:**
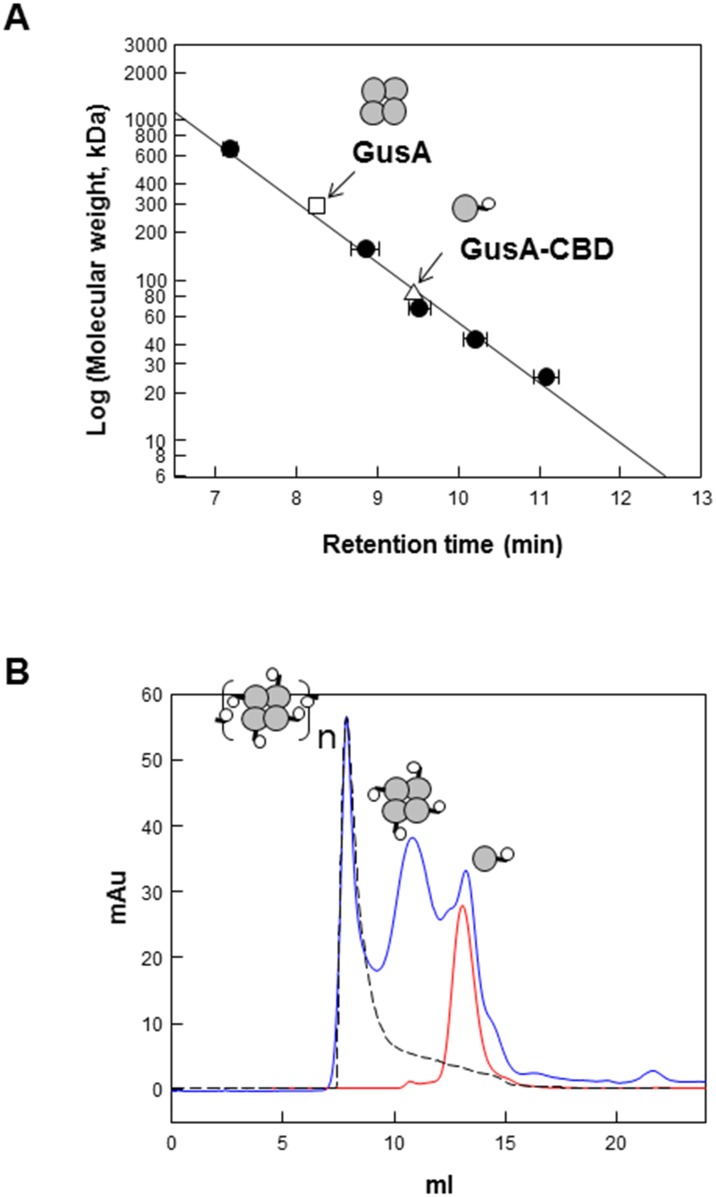
Determination of molecular mass of native GusA and GusA-CBD. (A) Determination of molecular mass of native GusA and GusA-CBD by using gel filtration chromatography. Thyroglobulin (669 kDa), aldolase (158 kDa), albumin (67 kDa), ovalbumin (43 kDa), and chymotrypsinogen A (25 kDa) were used as reference proteins (closed circles); GusA (open triangles); and GusA-CBD (open squares). Data represent the means of three experiments and error bars represent standard deviation. (B) Determination of native molecular weight of GusA-CBD (red line) and oligomer of GusA-CBD (blue line) after incubation for 12 h at 4°C. Blue dextran was used to determine the void volume (black dashed line).

The native molecular mass of GusA and GusA-CBD were estimated to be 290 and 83 kDa, respectively, via gel filtration chromatography by using a Superose_12 10/300 column (Fig 1A). The results indicated that native GusA exists as a tetramer, while GusA-CBD exists as a monomer. In addition, MALDI-TOF mass spectroscopy, which was used to confirm the native forms of the enzymes, showed the same results (data not shown), suggesting that CBD prevents protein oligomerization when fused to the C-terminus of the target enzyme.

CBD can be assembled into particles when purified CBD is placed in the proper solution. To determine whether the purified GusA-CBD can assemble into enzyme particles via the stickiness of CBD, the samples were incubated at 4°C for 12 h and then subjected to gel filtration chromatography in order to determine their aggregated forms. GusA-CBD was found to have the same retention time as blue dextran had, which was used to determine the void volume of the column (Fig 1B), indicating that the fusion protein aggregated as enzyme particles. Further analysis revealed that the native form was present under high temperatures and acidic conditions of 65°C and pH 5, respectively, for 20 min. The native form under various conditions was also detected in the void volume (data not shown). Oligomers should be further examined in detail via high-resolution microscopy. This suggests that the CBD-containing enzyme can exist in various states depending on the environmental conditions.

### Effects of pH and temperature

**Fig 2:**
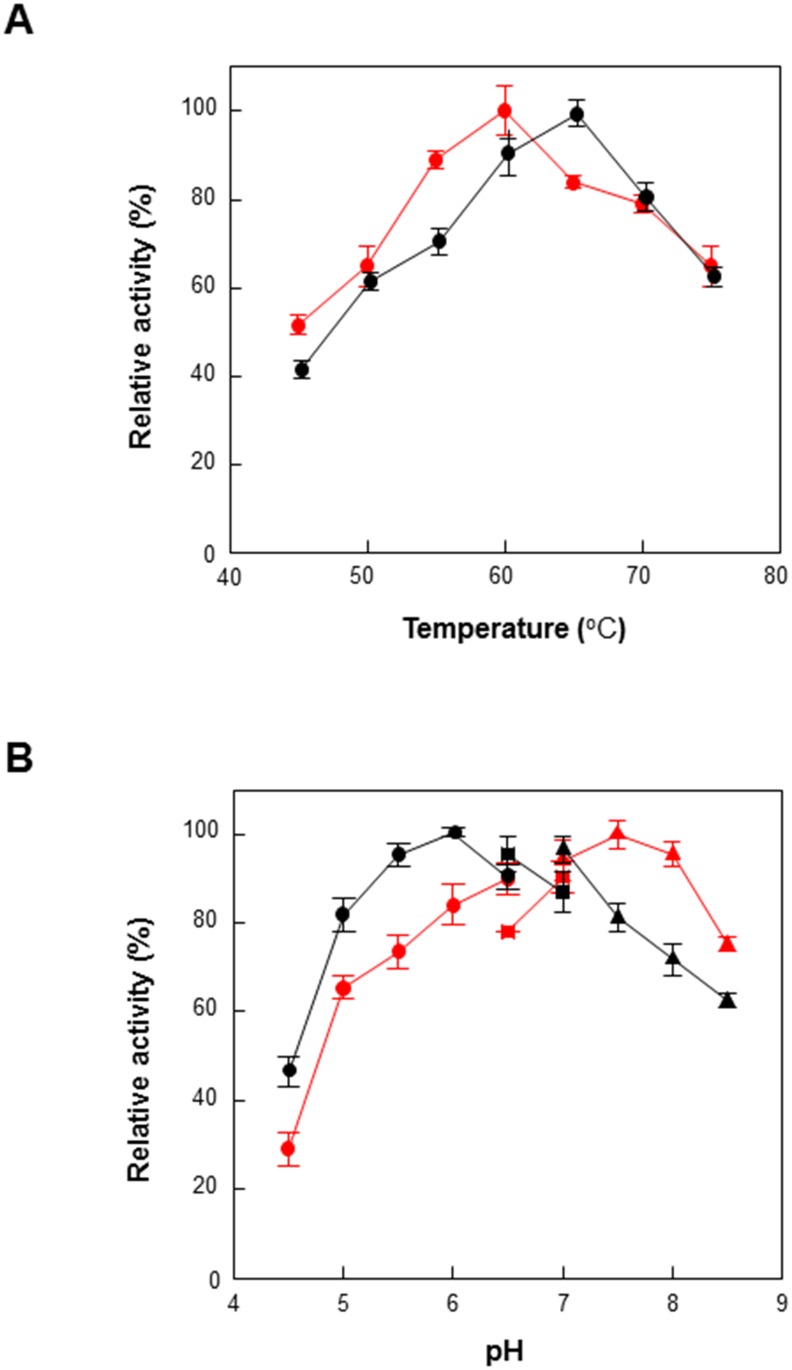
Effects of pH and temperature on enzyme activity of GusA (red) and GusA-CBD (black). (A) Temperature: Reactions were performed in 50 mM HEPES buffer (pH 7.5) containing 0.5 mM pNPGA, 0.2 U mL^-1^ of enzyme for 5 min. (B) pH: The reactions were performed in 50 mM sodium acetate (closed circles) or 50 mM K-phosphate buffer (closed squares) or 50 mM HEPES buffer (closed triangles) containing 0.5 mM pNPGA, 0.2 U mL^-1^ of enzyme at 60°C for 5 min. Data represent the means of three experiments and error bars represent standard deviation. The relative activities of 100% gusA and gusA-CBD were 102 ± 4.3 and 19 ± 0.6 U mg^–1^, respectively.

GusA is widely used a reporter enzyme because it can be easily assayed using a variety of commercially available chromogenic, colorimetric, and fluorometric substrates [7,8]. The optimal pH and temperature of GusA toward *p*NP-glucuronide were slightly changed when GusA was fused to CBD in the cytoplasm of *E*. *coli*. After purifying free GusA and GusA-CBD, the enzymatic activities were compared at different pH levels and temperatures with *p*NP glucuronide as a substrate. The maximum hydrolytic activity of free GusA was observed at 60°C and pH 7.5, while those for GusA-CBD were 60–70°C and pH 6.0, respectively (Fig 2A and 2B). Interestingly, GusA-CBD exhibited maximal activity at higher temperatures and acidic pH levels compared to free GusA. In a previous study, a family I carbohydrate-binding module of *Trichoderma reesei* cellobiohydrolase was fused with β-mannanase, resulting in an optimal temperature that was 5–8°C higher than that of free β-mannanase [13]. These results indicate that GusA-CBD is relatively stable at higher temperatures and more acidic pH values than free enzyme.

### Comparison of thermostability and pH stability

**Fig 3:**
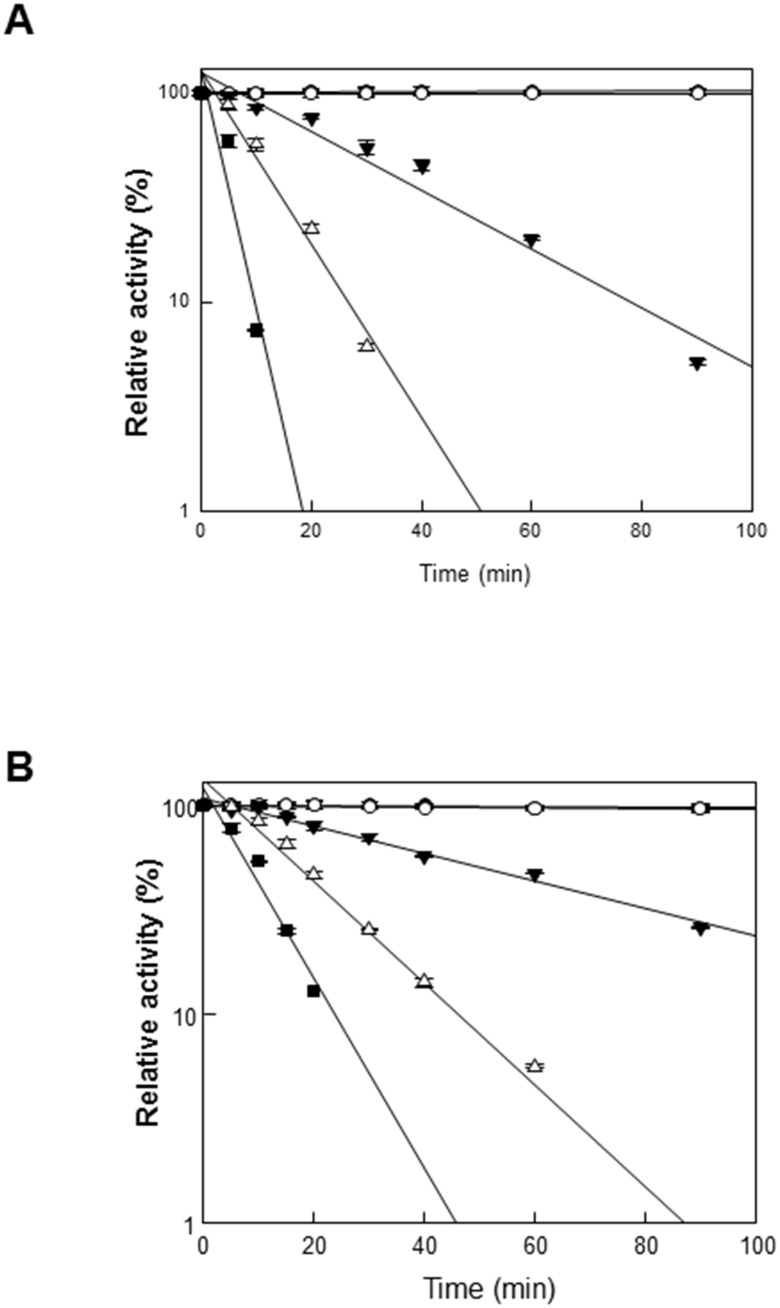
Thermal inactivation. GusA (A) and the GusA-CBD (B) at 40°C (closed circles), 45°C (open circles), 50°C (closed triangles), 55°C (open triangles), and 60°C (closed squares). Data represent the means of three experiments and error bars represent standard deviation. The relative activities of 100% gusA and gusA-CBD were 102 ± 4.3 and 19 ± 0.6 U mg^-1^, respectively.

**Fig 4:**
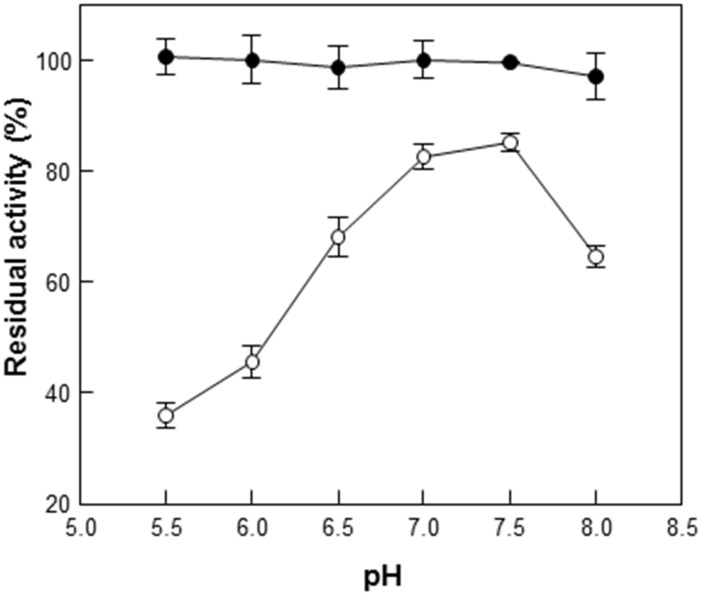
pH inactivation of GusA (open circles) and GusA-CBD (closed circles) at pH 4.5–8.0. Data represent the means of three experiments and error bars represent standard deviation. The relative activities of 100% gusA and gusA-CBD were 102 ± 4.3 and 19 ± 0.6 U mg^–1^, respectively.

Enzyme stability to heat and pH was also compared between free GusA and GusA-CBD. The thermal inactivation of GusA and GusA-CBD was evaluated at various temperatures. The half-lives of GusA at 45°C, 50°C, 55°C, and 60°C were 901, 28.5, 9.8, and 3.6 min, respectively (Fig 3A). However, those of GusA-CBD at 45°C, 50°C, 55°C, and 60°C were 1442, 51, 17, and 8.3 min, respectively (Fig 3B). Moreover, GusA-CBD retained 100% of its activity after 12 h at various pH values, whereas GusA retained less than 40% of its activity after 12 h at pH values below 5.5 (Fig 4). These results demonstrate that GusA-CBD has higher thermal and pH stability than GusA.

### Methanol tolerance and protease accessibility

**Fig 5:**
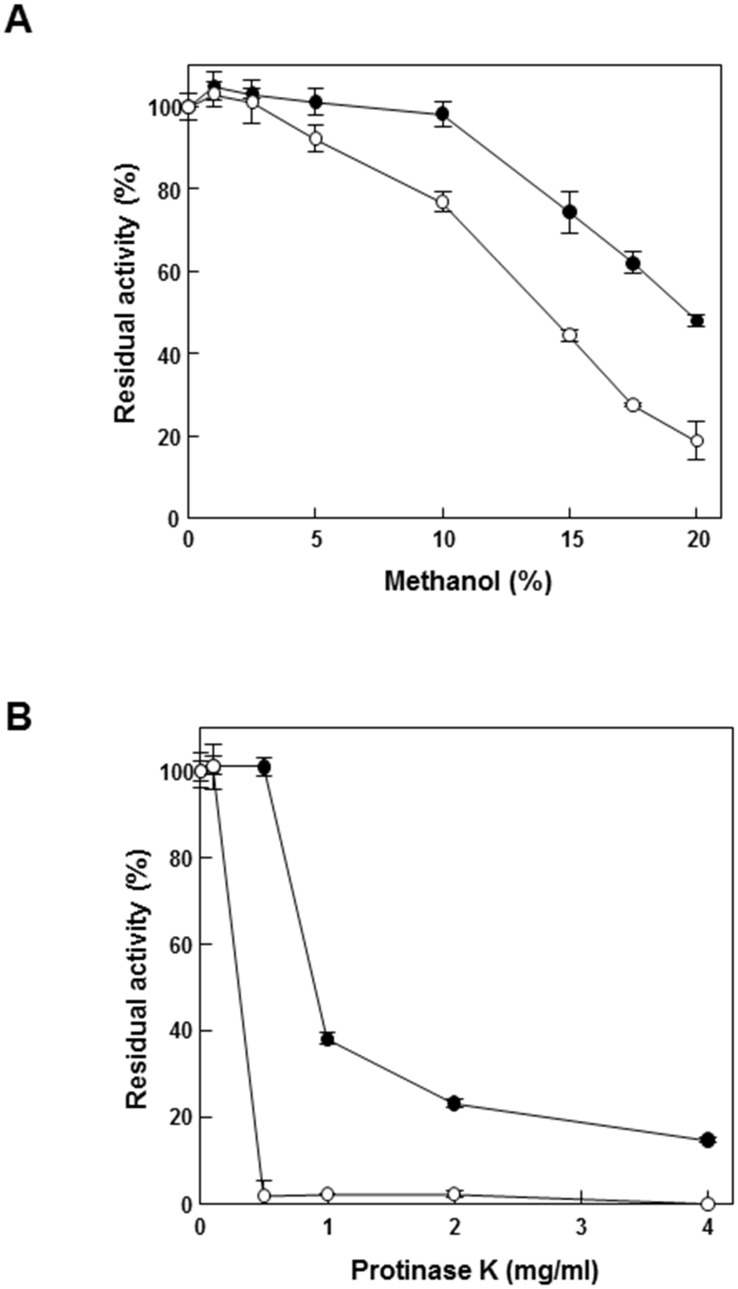
Tolerance test against methanol and protease K. (A) Effect of various concentrations of methanol on the activity of GusA (open circles) and GusA-CBD (closed circles). (B) Protease K accessibility on the activity of GusA (open circles) and GusA-CBD (closed circles). Data represent the means of three experiments and error bars represent standard deviation. The relative activities of 100% gusA and gusA-CBD were 102 ± 4.3 and 19 ± 0.6 U mg^–1^, respectively.

The solvent stability of enzymes is important for industrial applications. To test the solvent stability of GusA-CBD, methanol was selected. Methanol tolerance was determined at various concentrations of methanol from 0 to 20% (v/v) in the reaction mixture. As a result, GusA-CBD showed 30% higher activity than free GusA at different methanol concentrations (Fig 5). To determine the protease accessibility to GusA-CBD, protease K concentrations ranging from 0 to 4 mg/mL were added to the enzyme solution. When more than 0.5 mg/mL of protease K was added, free-form GusA completely lost its activity, whereas GusA-CBD retained its activity (Fig 5). This suggests that CBD prevented protein degradation against protease by concentrating the target enzymes, which is similar to the immobilization of enzymes to microporous resin.

## Discussion

Enzyme stability is a significant concern for protein engineers in the field of biotechnology because of its industrial importance. Numerous studies have reported methods for increasing enzyme stability while retaining functional activity via enzyme evolution [14–16]. *Escherichia coli* GusA has been engineered with improved stability and activity via various technologies, such as directed evolution, site-saturation mutagenesis, and DNA shuffling, because of its commercial potential [17–20]. Despite these trials for the evolution of GusA, false-positives were still observed because of endogenous GusA activity in the screening system [8]. Moreover, these screening systems require considerable time, labor, and optimized high-throughput screening methods.

In this study, we simply fused CBD to GusA and purified the resulting protein to generate an oligomeric enzyme that retained the activity of GusA while increasing the stability under various pH and temperature conditions (Figs 3 and 4). Moreover, GusA-CBD showed a high tolerance to organic solvents and protease (Fig 5). These results demonstrate that CBD is a useful matrix for enzyme stability. Depending on the experimental conditions (high temperature and acidic pH), soluble GusA-CBD was detected in the oligomeric state by size-exclusion chromatography, while free GusA was always tetrameric.

Increasing the stability of GusA may eliminate false-positives and dramatically increase its veracity as a reporter in high-throughput screening systems [10]. Previously, some shuffled GusA mutant variants were reported to be more thermostable than wild-type GusA [20], suggesting that quaternary structural changes such as oligomerization of mutant GusA increase enzyme stability because wild-type GusA quickly became completely monomeric at temperatures above 50°C. However, all thermostable mutant variants again became monomeric at temperatures above 70°C [20]. In this study, CBD-fused GusA retained its oligomeric state at temperatures above 70°C. These results suggest that the oligomeric state of the active aggregate using CBD will help to increase GusA stability, as observed in our *in vitro* immobilization analysis. By controlling the environmental conditions, the enzymatic state can be tuned to the desired application. In our experiment, we determined that CBD existed as an insoluble oligomer (in the whole cell), soluble monomer (at low temperature), and soluble oligomer (at high temperature and acidic pH) depending on the experimental condition (S2 Fig). First, this can be used as an enzyme immobilization matrix in *E*. *coli* cells by localizing cytosolic proteins to CBD particles [5]. Second, soluble CBD can be used as a matrix for biocatalysis *in vitro*. Third, soluble oligomeric CBD was detected under high temperatures and acidic pH conditions, which can help to improve enzyme stability.

Another example of the applications of CBD have been reported as active IBs [5,6] which show high levels of catalytic activity. It have shown several advantages such as easy purification and separation, greater stability, and good usability for applications including immobilized biocatalysis, bioassays, and biomaterials [21,22]. The enzyme activities, solubility, ratio in the cell, and selection of linker for design of fusion protein have not been widely examined [22]. Additionally, constructing synthetic scaffolds using the CBD-based system may further improve the metabolite conversion rate by increasing local enzyme concentration and reducing intermediate loss caused by diffusion or side reactions [5]. For example, the fusion of various CBD molecules to the N- or C-termini of other enzymes may enhance enzyme catalytic efficiency by increasing their local concentrations around the polysaccharide substrates such as insoluble cellulose, and/or improve catalytic activity and thermostability [13,23].

Consequently, the use of CBD as a simple fusion domain may be useful in synthetic matrix for the biochemical production of stabilized enzymes. Further studies using valuable bio-based chemical producing enzymes should be performed to examine the stability and maintenance of the activity of CBD-fused enzymes for bioprocess applications.

## Conclusion

In this study, we found that CBD existed in various enzymatic states, such as insoluble oligomers, soluble monomers, or soluble oligomers, according to the experimental conditions. Using this unique domain, the GusA-CBD enzyme showed increased enzyme stability under high heat, high pH, methanol, and protease, which are beneficial properties for industrial applications. This method may be applied in high-throughput screening or biochemical production of stabilized enzymes.

## Supporting Information

S1 Fig Determination of molecular mass of GusA and CBD fused GusA. (A) SDS-PAGE analysis of purified enzymes from each purification steps. Lane 1, crude extract of GusA; lane 2, IMAC column product (purified GusA enzyme) Lane 3, crude extract of GusA-CBD; Lane 4, IMAC column product (GusA-CBD); Lane 5, crude extract of CBD-GusA; Lane 6, IMAC column product (purified CBD-GusA enzyme); M, prestained marker proteins.
(TIF)

S2 Fig Schematic representation of enzyme catalysts with self-assembled CBDs. The GusA (yellow)-CBD (grey) complex with flexible linker was present in three states depending on the environmental conditions.
(TIF)

S1 Table Primers used in this study. (TIF)

S2 Table The specific activity of GusA, GusA-CBD, CBD-GusA. (TIF)
