# Supplementary figures and images for: Controlled Aggregation and Increased Stability of β-Glucuronidase by Cellulose Binding Domain Fusion
Source: PLoS One. 2017 Jan 18;12(1):e0170398. doi: 10.1371/journal.pone.0170398 (PMC5242468; doi:10.1371/journal.pone.0170398)

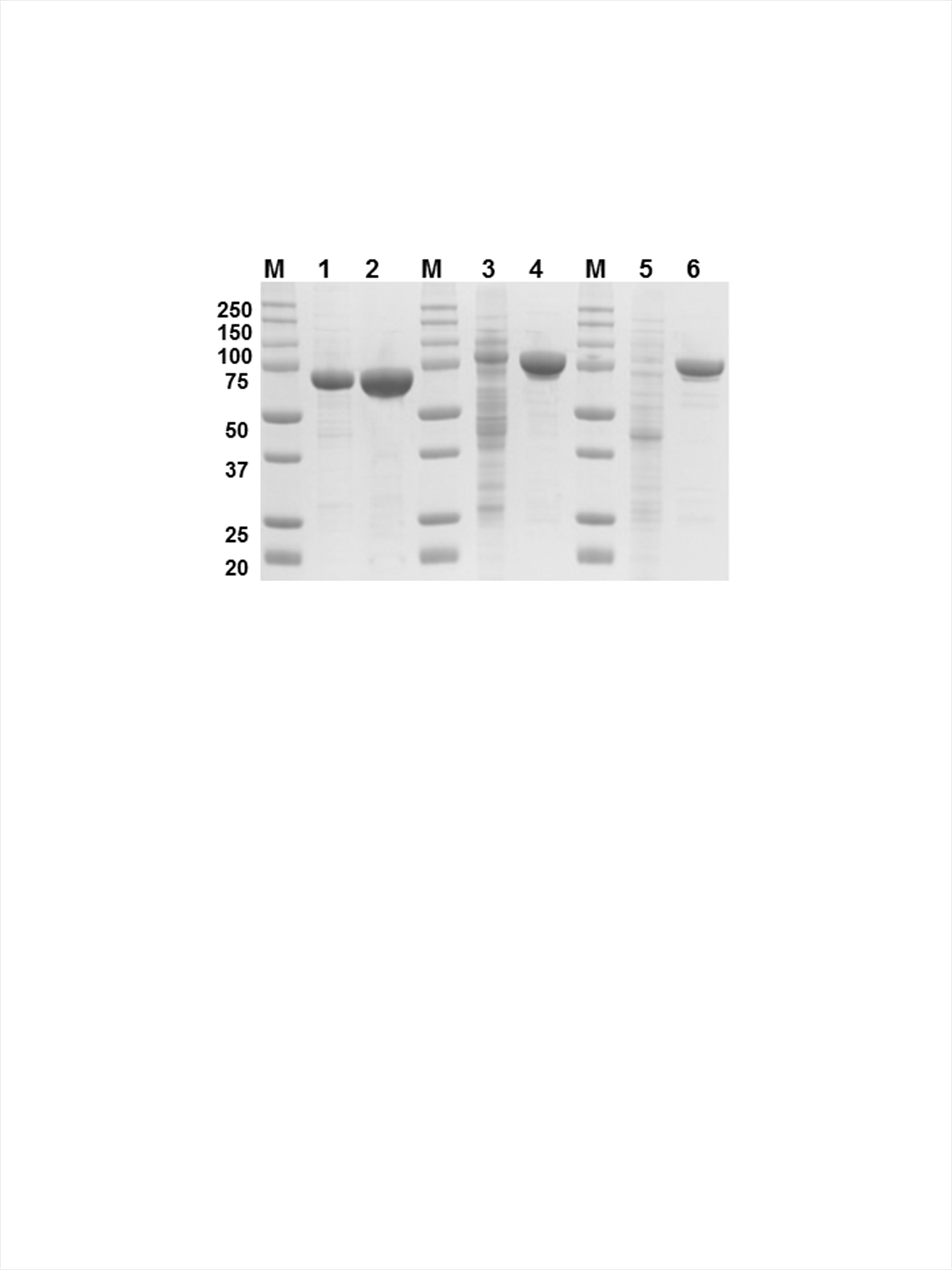

Supplement: S1 Fig — (A) SDS-PAGE analysis of purified enzymes from each purification steps. Lane 1, crude extract of GusA; lane 2, IMAC column product (purified GusA enzyme) Lane 3, crude extract of GusA-CBD; Lane 4, IMAC column product (GusA-CBD); Lane 5, crude extract of CBD-GusA; Lane 6, IMAC column product (purified CBD-GusA enzyme); M, prestained marker proteins. (TIF) [file pone.0170398.s001.tif]

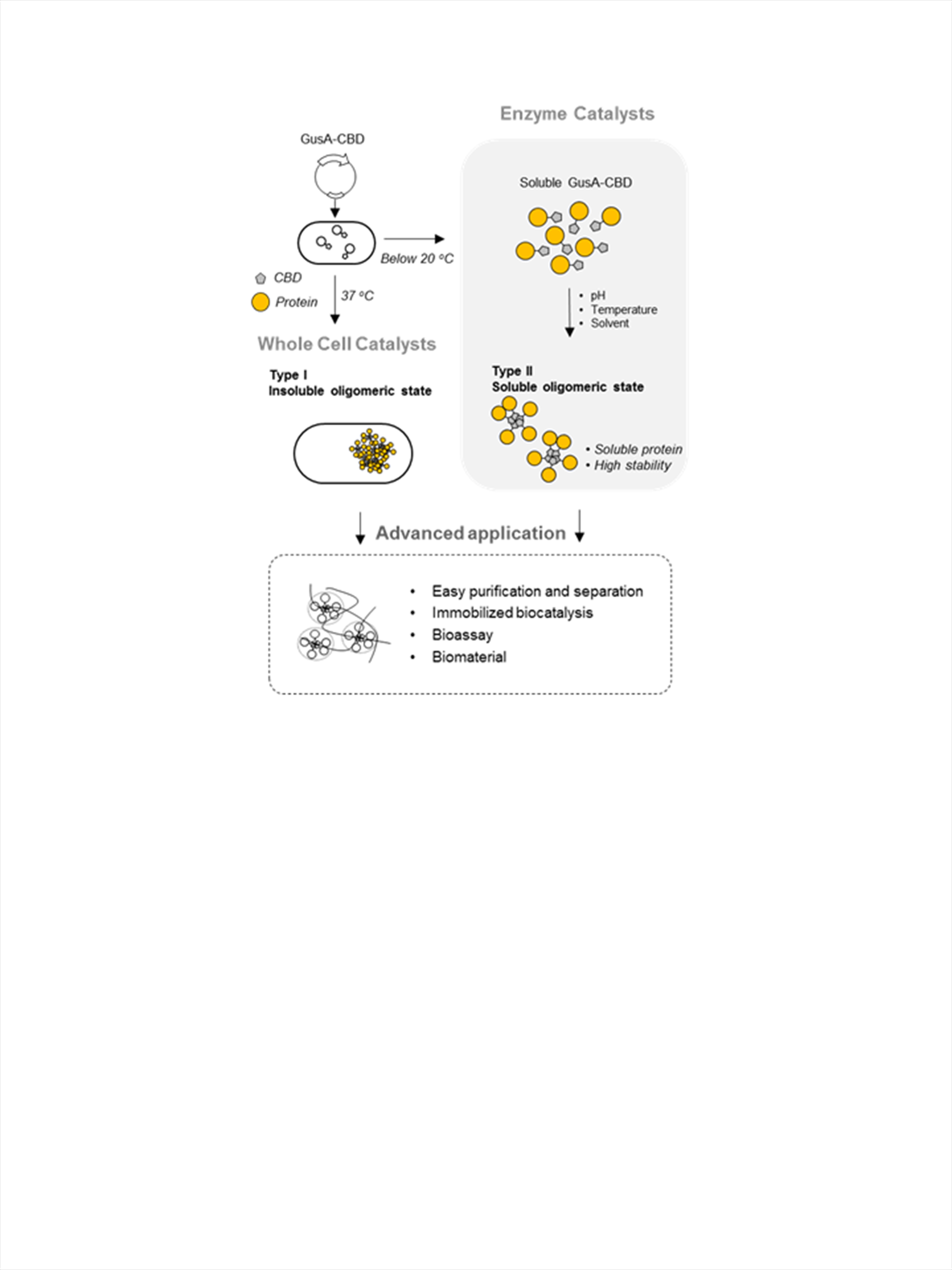

Supplement: S2 Fig — The GusA (yellow)-CBD (grey) complex with flexible linker was present in three states depending on the environmental conditions. (TIF) [file pone.0170398.s002.tif]

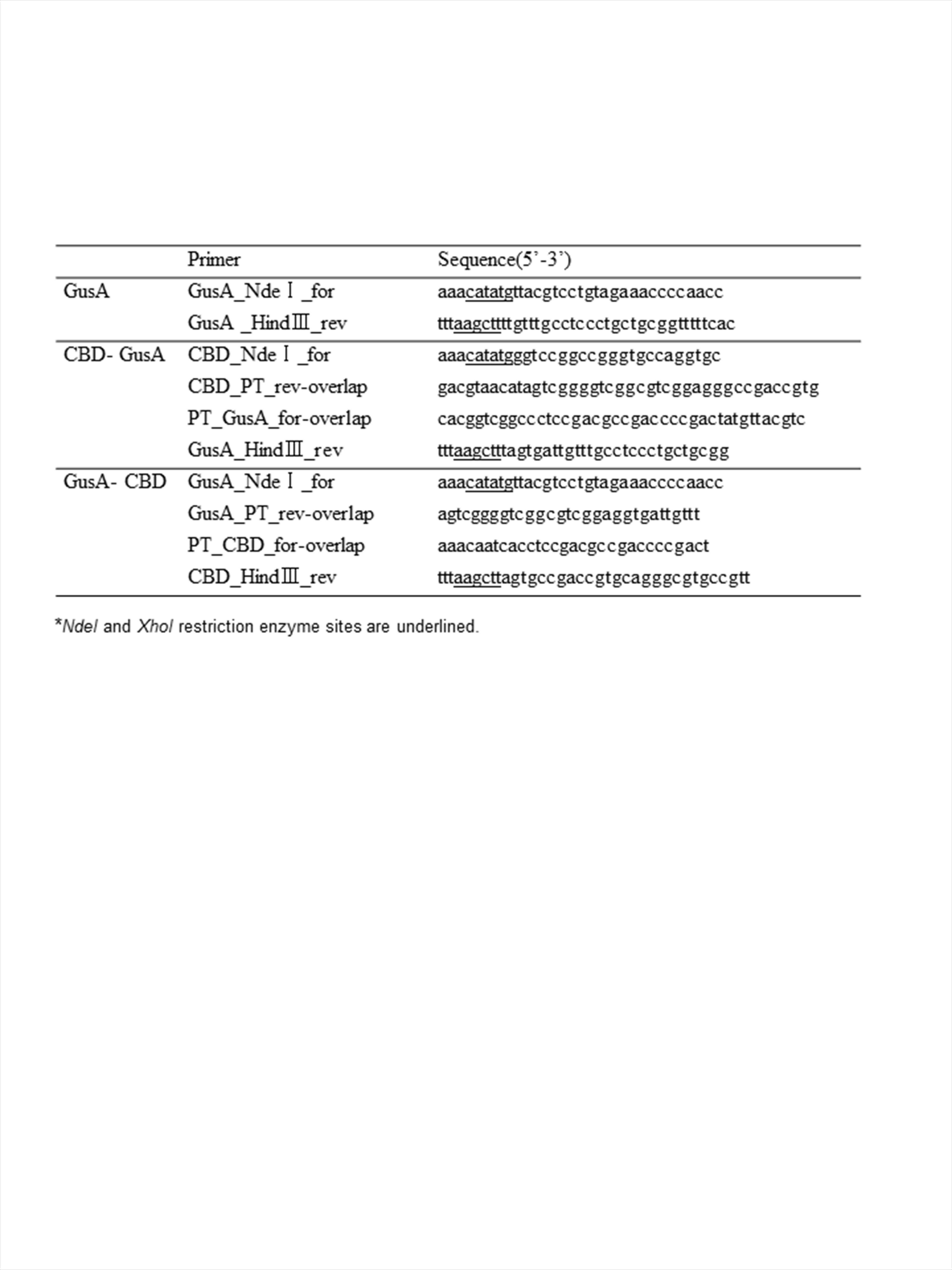

Supplement: S1 Table — (TIF) [file pone.0170398.s003.tif]

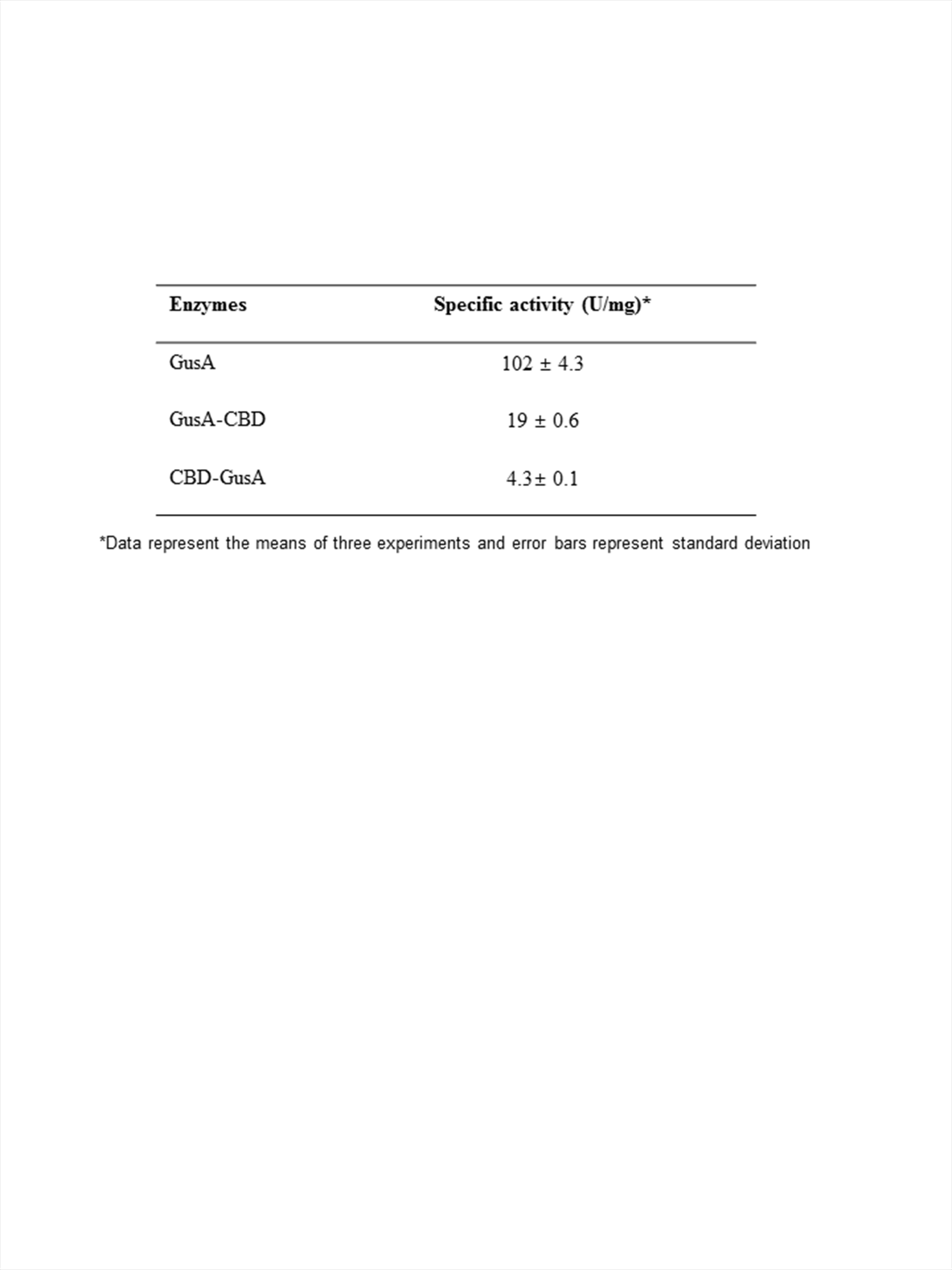

Supplement: S2 Table — (TIF) [file pone.0170398.s004.tif]
